# Distinctive nursing practices in working with mothers to care for hospitalised children at a district hospital in KwaZulu-Natal, South Africa: a descriptive observational study

**DOI:** 10.1186/s12912-020-00421-1

**Published:** 2020-04-19

**Authors:** Natasha North, Angela Leonard, Candice Bonaconsa, Thobeka Duma, Minette Coetzee

**Affiliations:** Child Nurse Practice Development Initiative, Department of Paediatrics and Child Health, University of Cape Town, Red Cross War Memorial Children’s Hospital, Klipfontein Road, Rondebosch, Cape Town, South Africa

**Keywords:** Nursing, Children, Family, Qualitative research, Visual research methods, South Africa

## Abstract

**Background:**

The presence of family members and their active involvement in caring for hospitalised children is an established practice in many African paediatric settings, with family members often regarded as a resource. This aspect of African paediatric nursing practice lacks formal expression or a clear conceptual basis, and difficulties arise when applying concepts of family involvement originating from the culturally distinct practice environments of higher resourced settings including Europe and America. The aim of this study was to articulate a nurse-led practice innovation intended to facilitate family involvement in the care of hospitalised children, observed in a paediatric inpatient ward in a district hospital in rural KwaZulu-Natal, South Africa.

**Methods:**

A qualitative case study design was used. Data collection included visual research methods (graphic facilitation, sociograms and photo-elicitation) as well as a focus group, interviews and practice observation. Activities associated with 20 nurses and 22 mother-child dyads were observed. Data were subjected to content analysis, with Standards for Reporting Qualitative Research (SRQR) applied.

**Results:**

Findings relate to six aspects of practice, categorised thematically as: preserving the mother-child pair; enabling continuous presence; psychological support and empathy; sharing knowledge; mothers as a resource; and belief and trust.

**Conclusion:**

The nursing practices and organisational policies observed in this setting relating to the facilitation of continuous maternal presence represent a distinctive nursing practice innovation. This deliberate practice contrasts with models of care provision which originate in higher resourced settings including Europe and America, such as Family Centred Care, and contrasts with informal practices in local African settings which tolerate the presence of mothers in other settings, as well as local institutional policies which limit mothers’ presence to varying extents.

## Background

In most cultures around the world, families are regarded as an essential resource in the care of the hospitalised child. The expectation that a family care-giver (usually the child’s mother or another female relative) will be continuously present alongside the child and will be responsible for some degree of practical care provision is a documented feature of paediatric care in Africa [1–3], Eastern Europe and Asia [4–6].

Studies of family involvement practices in paediatric inpatient facilities in Malawi [2, 7] and Kenya [8] have concluded that nurses lack a basis for sound implementation resulting from the absence of formal practice guidelines and institutional policy norms. In addition to noting the absence of practical guidelines, studies examining family involvement in hospitalised children in Africa’s paediatric care facilities have encountered difficulties when applying concepts of family involvement originating from the higher-resourced and culturally distinct practice environments of higher resourced settings including Europe and America [2, 8, 9], with international dialogue highlighting the differences in context and perspective between practitioners from different geo-cultural contexts [10].

While the practices and conceptual bases of enrolling families in the care of hospitalised children in Africa’s paediatric care facilities share some similarities with models of care provision which originate in Europe and America, such as Family Centred Care [11, 12], they are in important respects distinct. Descriptions of family involvement in caring for hospitalised patients in Africa and elsewhere suggest that care-givers may variously be responsible for maintaining the patient’s comfort, hygiene, wound care and monitoring the patient’s condition as well as providing food, linen, medical supplies and medication [1–3, 5, 13]. Makworo [8] documented that women remained the primary caregiver for their children even when the child was admitted to a paediatric facility in Kenya.

These practices are often explained in terms of resource scarcity [13], but we believe this explanation is incomplete and restricts fuller examination of the nursing knowledge and values which underpin these practices. It may not be the case, for example, that mothers are involved in care provision purely or mainly because of the comparatively low numbers of nurses available. Appropriate practices which do not conform to the ‘good’ practice or contexts that are taught and assumed in most nursing education often remain unacknowledged [14]. Nursing knowledge is socially embedded [15] and is easily made ‘invisible’ through the assertion of different social and cultural values [16]. The development of Afrocentric nursing practice models and tools is important in supporting evidence-based safe nursing practice [17], but researchers must find methods which enable the identification and description of promising local practices.

The purpose of the study described in this paper was to observe, describe and articulate contextually specific nursing practices in relation to facilitating family involvement in the care of hospitalised children in a paediatric inpatient ward in a district hospital in rural KwaZulu-Natal, South Africa. Specific aims were to:

- Identify explicit nursing practices and formal policies or guidelines associated with mothers’ presence in this setting
- Identify and describe implicit nursing practices associated with mothers’ presence in this setting
- Facilitate articulation by nurses of the rationales and values underpinning their explicit and implicit practice in relation to facilitating the continuous presence of mothers in this setting.

This study is part of a larger qualitative study using an instrumental collective case study approach to observe and document children’s nursing practice in relation to family involvement in the care of hospitalised children.

### Terminology

Whilst in many cases the women referred to as ‘mothers’ were the biological mothers of the children they accompanied, it seems that the definition applied by nurses was a functional one, related to the woman’s role in child-caring rather than child-bearing. Mother is therefore used to refer to any woman accompanying and caring for a child in this setting, whether or not they were the child’s biological mother or a grandmother, aunt, older sister or foster mother. No men undertook this role in this setting.

## Methods

### Research design

A qualitative case study design was used. Data collection included the visual research methods of graphic facilitation, sociograms and photo-elicitation implemented concomitantly with interviews, a focus group, and practice observation to support iterative narrative data collection. Standards for Reporting Qualitative Research (SRQR) [18] and guidelines for reporting qualitative case study research [19] were applied to describing the design and results.

### Research setting

The study was conducted in a 22-bedded paediatric inpatient ward of a district level hospital, in a remote rural area of the Umkhanyakude health district, in northern KwaZulu-Natal, South Africa. A descriptive summary of the salient contextual factors is provided in the results (see Table 1).

**Table 1:** A descriptive summary of the salient contextual factors of the study setting in accordance with good practice reporting guidelines [18]

***Staffing***	The ward is managed by a nurse manager who is a registered nurse, with an additional specialist qualification in paediatric nursing. There was an average of five nurses on each observed shift.
***Language***	The majority of the population living in the Umkhanyakude health district speak isiZulu as a first language. Nursing staff speak isiZulu and English with one another, and often speak isiZulu with patients. Written records are maintained in English.
***Service capacity***	The 22-bedded ward admits patients for a variety of medical and surgical conditions ranging in acuity with two high-care beds and a 5-bedded isolation facility. Reasons for admissions include: burns; gastroenteritis; snake bites; poisoning; pneumonia; traffic accidents; seizures; malnutrition, and social admissions (children who have been abandoned).
***Ward environment***	The main part of the ward is open-plan with full-sized beds in rows along each side. The 18 full-sized adult beds with cot sides allow the mother to share a bed with her hospitalised child. There are four small cot beds for children who are receiving orthopaedic traction or who do not have a mother staying with them. Each bed is separated from the next by a locker and curtains that are rarely drawn by mothers or staff.

### Positioning of the researchers

The field research team comprised of three postgraduate qualified nurse researchers from the Child Nurse Practice Development Initiative, one of whom had experience of practising in a contextually similar facility. The working languages of the hospital were English and isiZulu. Two of the researchers spoke English and one spoke both English and isiZulu at the level of full professional proficiency. All the researchers had received training in relevant research techniques. The nurse manager of the paediatric ward was enrolled as the key informant and assisted in logistics and brokering trust between participants and researchers.

### Population and sampling

The total population for this study was all nurses working on the ward during the period of observation (*N* = 20), and all mother-child pairs present in the ward (*N* = 22). Sampling for inclusion in interviews and focus groups was intended to be as close to comprehensive as possible, with all nurses working on the ward during the period of observation and all mothers and children present on the ward eligible for inclusion, subject to consent.

### Data collection

Graphic facilitation [20–22], sociograms [23–25] and photo-elicitation [26–28] were used to stimulate participant engagement in individual and focus group interviews, with the intention of eliciting conceptually rich accounts of practice which were grounded in the cultures of the setting [29–31]. A detailed description of the participatory visual research methods employed has been provided elsewhere, together with outline interview schedules [31]. Table 2 summarises the process of iterative data collection using visual methods.

**Table 2:** Summary of the process of iterative data collection using visual methods

Activity	Visual method(s) used as stimulus	Purpose	Timing
Initial interview with nurse manager	*Photo-elicitation*	Generate a description of facility norms of practice, relating to the involvement of families in the care of their children. Begin to explore the rationale for practices.	After generating photographs, near the start of practice observation.
Focus groups	*Graphic facilitation*	Stimulate nurses’ narrative accounts of what happens to children and their families in this setting, and why. Generate a visual representation of the pathway of care, tracing children’s individual journeys into, through and out of the healthcare setting, identifying: the extent of family involvement at each stage; the nursing practices associated with family involvement, and the underlying rationale for nurses’ practices. Elicit nurses’ accounts of what they think and feel about involving families in caring for children.	At least two per site. One near the start of practice observation.
Individual interviews with nurses	*Graphic facilitation* *Sociograms* *Photo-elicitation*	Elicit nurses’ accounts of activities observed.	Ongoing throughout data collection.
Interviews with family members	*None*	Generate families’ accounts and explanations of nursing practices. Enable comparison of families’ and nurses’ descriptions of practice.	Ongoing throughout data collection. Summary added to graphic
Subsequent/final interview(s) with nurse manager	*Photo-elicitation* *Graphic facilitation* *Sociograms*	Refine the description of practices and explore inconsistencies arising from other accounts of practice e.g. focus groups. Further explore the rationale, philosophy and culture behind observed practices.	Close to the end of the period of practice observation.

Data collection took place over three consecutive days in September 2017. The focus group and all the interviews were audio recorded and transcribed verbatim, with interviews conducted in isiZulu translated into English during transcription.

### Trustworthiness

Credibility was maximised by using an iterative data collection research strategy. The researchers continuously invited comment on the interpretation of data and emerging insights from the nurses in the setting, working towards the development of a shared understanding. This supported triangulation as researchers were able to confirm or challenge emerging findings from multiple observations, interviews and the focus group, with participants in the field. Visual methods were supportive of this iterative approach to triangulation, since the same image/depiction of practice was subjected to multiple perspectives, identifying similarities and exploring inconsistencies. Researchers sought and documented feedback on interpretation of findings through member checking a draft report with the key informant [32–36] to further enhance validity. Transferability was addressed by the provision of a full description of the setting. To increase confirmability and dependability, the researchers maintained an audit trail of theoretical and process notes [37].

### Data analysis

Content analysis was conducted using the approach described by Erlingsson and Brysiewicz [38]. The data analysed were the transcribed records of focus groups and interviews. Transcribed material was read and re-read by all researchers to ensure familiarisation. Condensation of the text into meaning units was carried out with reference to the guiding questions (what are the nursing practices associated with mothers’ presence, and what rationales and values underpin these practices?). Initial codes were suggested by one researcher before discussion and refinement with two other researchers prior to adoption. Every data meaning unit was then coded by two researchers working independently. Where researchers did not agree on coding, the reasons for the discrepancy were discussed and a solution was agreed on, with revisions made to the code definitions if necessary. A third researcher was involved as necessary to help reach agreement. An example of the analysis process is provided in Table 3. Themes were formed after coding of all transcribed material.

**Table 3:** Example of the analysis process

Data extract	Initial code	Refined code	Preliminary theme	Main theme
*“The hospital management queried the mother staying with the child, so I said no this is the paeds ward, the mother and the baby need to stay together.”*	Nursing practices associated with mothers’ presence	Mothers who stay	Mothers who stay: b) why do they stay	***Preserving the mother-child pair***
*“The mother must see whatever we [nurses] do to the child and must master the care of the child that she would even be able to continue at home.”*	Underpinning rationales and values	Approaches to working with families to care for children	Equipping mothers to care	***Belief and trust***
*“It is also easy to observe if the mother is doing anything [not right] and then give education there and then and to create that bond with the child.”*	Nursing practices associated with mothers’ presence	What nurses do	Teaching and educating	***Sharing knowledge***
*“It is difficult to give medication to a child, it can take up to 15 min to give medication to one child, but with mother around it is so easy because the mother knows how to make their child to take medication, so it is working for [all of] us.”*	Underpinning rationales and values	What mothers do	Mothers as a resource	***Mothers as a capable resource***

## Results

Activities associated with 20 nurses and 22 mother-child dyads and two unaccompanied children were observed. Six mothers, two registered child nurses and two doctors participated in individual interviews while nine nurses (three registered nurses, five enrolled nurses and one enrolled nursing auxiliary) participated in the focus group. Six sociograms, 40 photographs and one graphic record were obtained.

### Explicit nursing practices and policies associated with mothers’ presence

Analysis of data enabled identification of a number of explicit nursing practices and policies associated with mothers’ presence in this setting, involving the following elements:

- An explicit expectation that a mother/grandmother will remain with the child throughout their hospital stay.
- Most mothers co-sleep with their child for the duration of their child’s hospital stay in full-sized beds, except in specific clinical situations, such as children who are receiving orthopaedic traction.
- Provision of meals for mothers at no cost to mothers.

These elements of practice are documented in a variety of ways, offering evidence that they represent formalised practice and organisational policy (see Table 4). A clear narrative account of the rationale for facilitating mothers’ presence was identified. The rural location means mothers often have to make long journeys to bring a child to hospital, expending significant resources. Nurses and mothers recognised that if mothers were not accommodated, they would have no choice but to return home and would then lack the resources to make return visits for follow-up care. This situation is common to many hospitals serving underserved rural communities, where the response is often to allow mothers to stay informally, or to provide a lodge or similar facility on site while permitting mothers’ presence on the ward during specified hours. The practice observed in this facility however adds a different dimension, moving from allowing mothers to stay, to making the continuous presence of mothers an explicit norm.

**Table 4:** Explicit nursing practices and policies associated with mothers’ presence

Observed practice	Formalisation through policy or resourcing	Explicit rationale	Initial code	Final main theme
The expectation that a mother/grandmother will remain with the child throughout their hospital stay is communicated to mothers on arrival at the hospital, or when they are referred from clinic.	The ward admissions policy states that a mother/grandmother should remain with infants and children under the age of 10 years for the duration of their hospital stay. The ward’s visiting policy differs from that of the rest of the hospital.	The ward’s visiting policy states that the policy is to promote unrestricted visiting to facilitate parental and family involvement.	Mothers who stay	***Enabling continuous presence***
Most mothers co-sleep with their child for the duration of their child’s hospital stay in full-sized beds, except in specific clinical situations, such as a child who is receiving orthopaedic traction.	A copy of an official notice explaining the practice of co-sleeping, signed by the hospital Paediatric Medical Officer and Ward Acting Nurse Manager, is displayed on the wall.	*“In 2005, when I first came to work here in the hospital from school health nursing, we only had the small cot beds and mothers were sleeping on mattresses on the floor. It was chaos”. (Nurse Manager, s21)*	Mothers who stay	***Preserving the mother-child pair***
The ward manager’s proposal to purchase 18 adult sized beds to enable implementation of a formal policy of co-sleeping for mothers and children was supported by hospital management.	18 adult sized beds with additional child-sized beds available if specific circumstances prevent co-sleeping	*“They changed that because the mothers were not comfortable as well as the babies, because they didn’t sleep together with their babies. The babies were sleeping on top and the mother’s underneath, and the babies were crying, and the mothers were taking their babies on the floor”. (Nurse, S6)* *“We supply the mums with big beds to sleep together with their child. ...A mother and child always sleep in the same bed.” (Nurse, s20)*	Equipment and facilities	***Preserving the mother-child pair***
Meals are delivered to the ward from the hospital kitchen and served to the mothers at the bedside.	The hospital provides three full meals a day for mothers and children at no charge.	*“They [general orderlies] bring the food from the main kitchen and dishes from here [ward kitchen] and serve the food to the mothers and children. The mothers get served breakfast, tea and bread, lunch and supper. There is a menu for every day, they get fish fingers, eggs, porridge and so on.” (Nurse, s20)*	Equipment and facilities	***Preserving the mother-child pair***

Table 4 shows how exploration of the explicit rationale for the formalised practices elicited data relating to initial codes of ‘mothers who stay’ and ‘equipment and facilities’. The decision to make formal provision for mothers was presented as a logical response. Nurses described the practical problems mothers encountered making return trips to the hospital, to the detriment of the child’s care, as well as the ‘chaos’ that resulted from accommodating mothers informally in the ward.

While the primary reason given for implementation of co-sleeping in this setting was a practical one, based on the need to accommodate mothers, analysis of data revealed the existence of other implicit practices, rationales and values related to the presence of mothers in this setting.

Six main themes relating to the practice of family involvement were identified (see Table 5). Findings deriving from observational data as well as interviews, field notes and photographs are presented in relation to each of the thematic headings, with an interpretation of the way the findings contribute to the development of the emerging concept of Care Through Family by nurses in African paediatric settings.

**Table 5:** Main themes of a Care Through Family approach to caring for hospitalised children

***Preserving the mother-child pair***	The goal is to ensure that the mother’s role in caring for the child continues with as little interruption as possible, with the exception of the medical event that has occurred. The normal place of care for the child is the home, and the family are their normal carers.
***Enabling continuous presence***	Policies and amenities are directed towards enabling the presence of mothers. Accommodation, space and amenities are organised to enable mothers’ continuous presence.
***Belief and trust***	Nurses and mothers have innate confidence in mothers’ abilities to learn and to cope, and high expectations about the speed at which they will become competent in new activities.
***Psychological support and empathy***	Enabling mothers to be physically and psychologically present and equipped to care involves empathetic practical and psychological support and the integration of social and psychological factors alongside physical care.
***Mothers as a capable resource***	Mothers are regarded as a resource within the healthcare system for their children in hospitals and at home by both nurses and mothers.
***Sharing knowledge***	The transmission of knowledge between nurses and mothers happens through ‘being with’ and ‘being taught’. The process through which mothers become competent to manage the child’s needs outside of hospital is dynamic, and responsive to the mother’s individual situation and progress.

### Implicit nursing practices and policies associated with mothers’ presence, and underpinning rationales and values

#### Preserving the mother-child pair

Although the majority of practices associated with facilitating mothers’ presence were quite tangible and therefore largely explicit, we also identified implicit rationales and values behind practical arrangements such as the provision of adult-sized beds, bed linen, and meals for mothers. Interviews and focus groups, stimulated by photographic interviewing in particular, revealed nurse participants’ sense of pride in being able to meet the needs of mothers and children during their stay, whilst recognising that not all facilities had access to the resources they had. Mothers are provided with hospital attire (known locally as ‘kitting’). The amenities on offer were clearly appreciated by mothers, as was the organisational culture of generosity.

*“We supply toilet paper and hand towels, even the nappies we supply for those babies who wear nappies.” (Nurse, s20)*

*“If you need anything then you could ask and I think that the nurses would give you. If you want to wash your clothes you can wash them and then take them to the laundry where they are dried and ironed. The laundry gives us clean hospital clothes every day.” (Mother, s13)*

Mothers recounted their experiences of accompanying a child for treatment at other hospitals with different policies regarding the presence of mothers:

*Mother: “Yes [she slept on the toddler sized bed] for three weeks.*

*Researcher: You can’t sleep in those little beds… so what did you sleep on then?*

*Mother: A coffee table [grimaces]. There’s a coffee table there. Because I cannot leave her alone.” (Mother, s16)*

This mother reported living more than 100 km from that hospital and lacked the resources to find accommodation in a town where she did not have family:

*“We went to [hospital A], we were there for four days. [Hospital A] is different because he sleeps alone in his bed and I sleep on the benches. You join the benches and then you sit next to your child and you sleep on them. They [the nurses] say they are doing you a favour by allowing you to sleep next to your child. You are not allowed to be with your child all the time, you can only come in at certain visiting times to see them. You were told to stay at home, where you normally stay. At [hospital A] there is no accommodation for mothers and that is why we sleep on the bench. They [nurses] say it is only children that are supposed to be here that is why we slept on the benches. Another thing at [hospital A] is that you are told as a mother you will not be given food. Mothers were not given meals, even if your home was far away you were still not given any meals”. (Mother, s15)*

#### Enabling continuous presence

We observed nursing care practices and interactions which suggest an implicit expectation that the mother should provide care for the child in the same way that she usually does at home. Mothers and children are essentially regarded by nurses as a single unit:

*“We promote a healthy whole for the child. If the child is alone, they cry, they do not eat and so we allow the mothers to stay together with their child. It is easy to heal faster with a mother”. (Nurse, s20)*

*“We need the mother and baby sharing the same bed like at home, so that the hospital environment cannot differ that much from home environment”. (Nurse Manager, s21)*

The policy of continuous maternal presence enables the mother’s role as the child’s primary caregiver to continue uninterrupted. Nurses preserved the mothers’ role as the primary provider of hands-on basic care for their child without interruption. Only in the absence of a mother would a nurse ‘take over’.

*“If the mum is not here, nurses take over, look after the patient. We are feeding them, bathing, because there is no mum”. (Nurse, s6)*

Mothers provide almost all the hands-on care for their child, adapting ordinary caring practices in response to the hospital environment or the child’s altered medical needs (e.g. tube feeding or mobilising after orthopaedic surgery) as an extension of their usual role:

*“I bath him, and I make sure that where he is playing is safe and that he's not going to hurt himself. I wake him up to give him his medications. Even if he doesn't want to eat, I am able to encourage him, and I feed him patiently”. (Mother, s13)*

*“I must help her. I just carry her and put her down and help her to walk.” (Mother, s16)*

Data from two direct observations emphasise the degree to which the presence of the mother comforts the child and the ease with which care continues:

*Child is sat against grandmother in bed, appears entirely relaxed throughout and does not object to presence of the doctor, medical student, nurse and observer. (Direct Observation)*

*[On completion of the dressing change] The mother immediately put the baby to the breast while she was still standing, and quickly moved to lay on the bed and continue breastfeeding. The baby settled instantly, mid cry. (Direct Observation)*

#### Belief and trust

Nurses in this setting trusted mothers to be responsible for aspects of their child’s care. While the child was in hospital nurses expected mothers to participate in care such as observing the child’s condition and reporting changes and concerns, assisting with prescribed physiotherapy exercises, providing a reassuring presence for the child during procedures and dressing changes, and assisting with giving medication.

Observation of nursing care practices in this setting suggested that both nurses and mothers have innate confidence in mothers’ abilities to learn and to cope, and high expectations about the speed at which they will become competent. Practices such as tube feeding were regarded by nurses as straightforward tasks that mothers could quickly become familiar with following minimal instruction, and observations of mothers who were tube feeding babies suggested that mothers were comfortable and exhibited no anxiety.

*I [researcher] asked the nurse in charge if this was normal practice [mothers to tube feed their child] and she said ‘yes’. If a child needs to be tube fed, the mother is taught to tube feed her own baby. (Direct Observation)*

Nurses’ accounts suggested that they regarded the presence of mothers as supporting the smooth running of the ward, reducing demands on nurses and contributing to faster healing and recovery for the child. Nurses were observed coming alongside mothers to provide information and feedback in a way that upheld the mother’s position as the child’s main carer. This was seen as having benefits during the period of hospitalisation and beyond, for both the child and the nursing staff.

*“...with mum around it is so easy because the mother knows how to make their child to take medication, so it is working for [all of] us”. (Nurse Manager, s21)*

*“[Mothers chose to stay] Because they love their child. And the babies also understand more of their mothers than with other people. Even with the medication, the babies will take it more easily with the mothers than with us.” (Nurse, s6)*

*“So, it is positive, so the mothers have jobs to do [breastfeeding] and even the changing of the nappies”. (Nurse, s2)*

Mothers indicated that they were aware that nurses continued to supervise some aspects of care, and nurses articulated their rationale for maintaining oversight in specific situations:

*“But, you know mothers, they sometimes cheat when they want to go home and say that the stools are normal but we [nurses] need to check. The reality is that we need to witness the stools… especially in the babies with gastroenteritis”. (Nurse Manager, s21)*

#### Psychological support and empathy

**Fig. 1:**
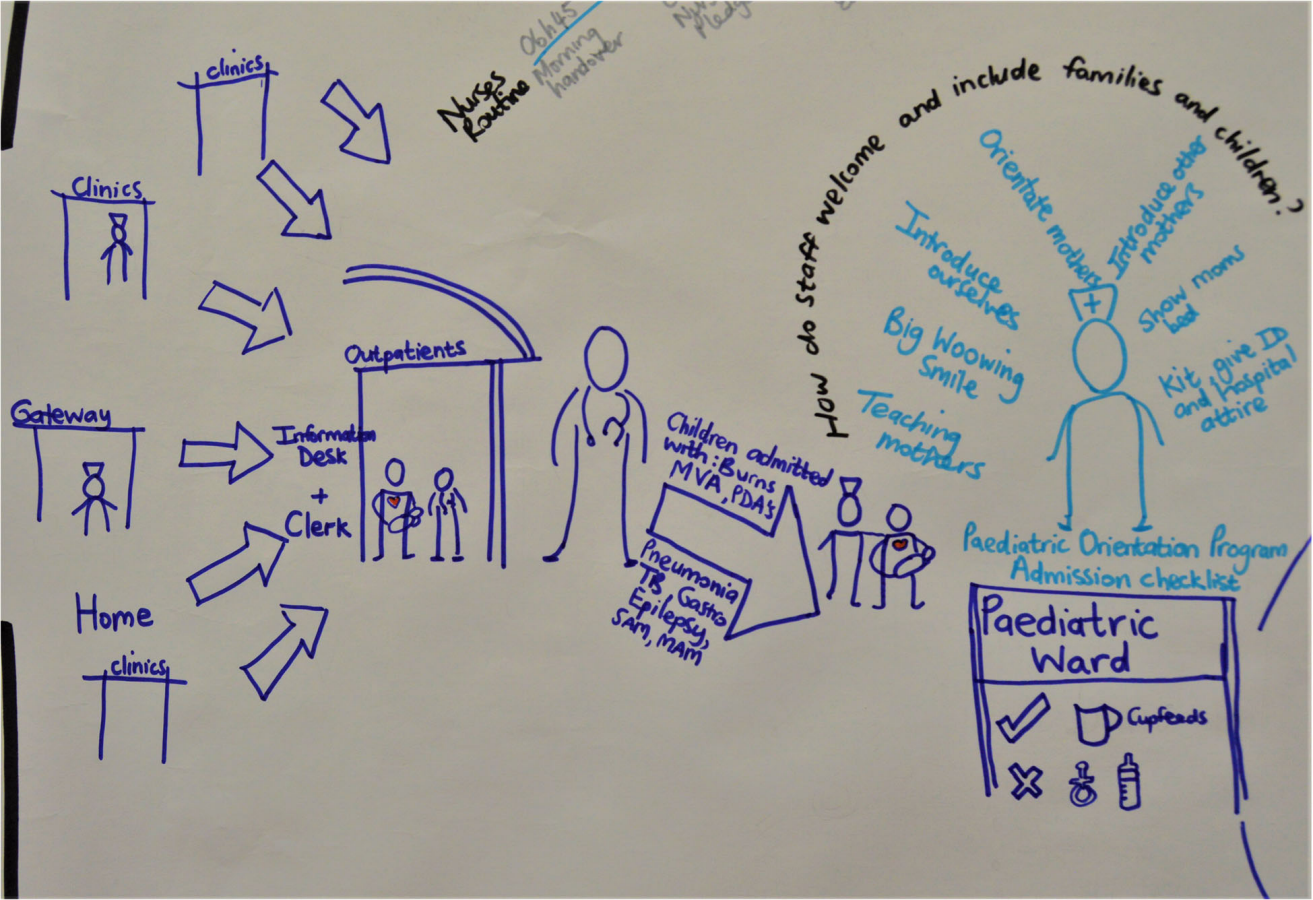
Making mothers welcome

Nurses described an authentic intention to provide care aimed at promoting the physical, social, emotional and psychological well-being of the mother and child. The rationale for the carefully considered ward policies and processes already described extends beyond making practical provision for mothers’ presence in the ward. The descriptions of practice stimulated by graphic facilitation suggested an emphasis on ‘welcoming’ mothers to the ward (see Fig. 1).

Nurses’ accounts of practices revealed that they are designed to enable the mother to be physically close to and emotionally and mentally present for her sick child. Amenities ensure that all her physical needs are taken care of, while a relaxed ward atmosphere with minimal routines reduces anxiety and frees her to focus on her child.

*Mothers are asleep in their beds in the middle of the day, there is no specific routines for mothers, other than having a bath or shower early in the morning. (Direct Observation)*

Observation data describes mothers being served courteously by domestic staff and treated with dignity and respect in all interactions with staff. There was a sense that mothers were cared for in ways that went far beyond simply tolerating their presence. Nurses are interested in and actively responsive to a mother’s social and emotional wellbeing and health needs. Nurses ensure that, where possible, these needs are addressed appropriately. It is as though, in viewing the mother and child as a single unit, nurses accept that caring for the mother is part of their responsibility.

*“Sometimes the mother comes here without their own treatment…then we ask the doctor to write a new prescription and order the treatment for them. We ask the mother about social problems…so we can pick up social problems, we then tell the doctor and they refer to the social worker”. (Nurse, s2)*

#### Mothers as a capable resource

In this setting it was striking to observe the way that mothers exhibited a relaxed sense of ‘belonging’ within the communal ward environment. Direct observations suggested there was a sense of community among the mothers who ‘room-in’ for the duration of their child’s hospitalisation. Overall, mothers appeared comfortable and at ease in the ward environment, with nurses unobtrusively facilitating this through the ward routine and their interactions with mothers, rather than formalised arrangements such as ‘support groups’.

Mothers were spontaneously described by nurses in ways that suggested nurses regarded their continuous presence as an important resource:

*“Mothers can do the feeding while we are busy with the doctors in the ward and doing procedures. Working together with mothers assists us in speedy recovery of patients”. (Nurse, s6)*

*“If the child is alone they cry, they do not eat and so we allow mothers to stay together with their child. It is easier to heal faster with a mother”. (Nurse, s20)*

Mothers indicated awareness of the extent to which nurses regarded them as a resource, and appeared to accept the responsibility without question and indeed to regard it positively:

*“I am in hospital so that I can be close to her and look after her, because nurses cannot always be with my child. Also, so that I can see if there is something not going well with my child and tell the nurses”. (Mother, s14)*

It was rare to hear a child crying or exhibiting signs of distress. During the period of observation, a variety of procedures were observed. In these cases, the mother was central to providing reassurance and comfort and was given a prominent role in the procedure by nurses:

*The mother was holding the child while the nurse cut off part of the burns dressing. The mother lay the child down on the bed, which was her normal bed in the ward, while the dressing was cleaned, and the mother consoled the child by rubbing the child’s arm and head. When the dressing had been changed, the mother picked the child up immediately and the child was consoled. (Direct Observation)*

Mothers appeared to give and receive both practical and emotional support to one another, and to one another’s children. Mothers were observed participating in caring activities for children other than their own, for example pouring juice and responding to requests for help, such as to pass a set of crutches.

Beyond the provision of practical support, nurses indicated that they regarded mothers providing psychological support to one another as a valued resource and indicated that they regarded interaction between mothers and the sharing of experiences and stories as beneficial. Providing psychological support was not the sole preserve of nurses:

*“We give them [mothers] psychological support and let them talk to other mums, sometimes other mums have the solutions to each other’s problems”. (Nurse, s4)*

#### Sharing knowledge

The data extract presented in Table 3 shows that the ability to teach mothers is a part of the explicit rationale for their presence in this setting. However, nurses’ accounts also pointed towards implicit ways in which the continuous presence of mothers was integral to the way nurses in this setting work to share knowledge. Mothers were expected by nurses to become competent at managing the child’s health needs through a dynamic two-way process of knowledge sharing and nurses exhibited a belief that mothers had deep understanding of their own children*.*

The mothers’ continuous presence was seen as making it possible for learning to take place more effectively than would otherwise have been the case, working towards the goal of the child and mother returning home with enhanced health capacity. Vicarious learning in this setting is facilitated by nurses ‘there and then’ in a responsive and opportunistic fashion, driven by the needs of the mother and child, and the opportunities afforded by daily events:

*“We give education about the child’s diagnosis on admission, we check in the file what the doctor wrote as the diagnosis...we tell the mother about the sugar salt solution. We do that there and then. We give education according to the child’s diagnosis”. (Nurse, s11)*

Opportunities to share knowledge written in the local language were integral to the fabric of the ward.

*“Here are the teachings on the wall written in isiZulu. It is the oral rehydration method with pictures to reinforce the message to mothers. It is to remind mothers about the oral rehydration solution”. (Nurse, s20*).

Nurses were also observed employing formal instruction one to one with mothers or gathering small groups of mothers in the ward setting to provide health education sessions. Topics and practices included provision of basic health education advice regarding infection prevention and control, including hand hygiene, practical steps within the home to reduce the risk of accidents such as burns, and the correct management of acute gastrointestinal illness, including preparation of oral rehydration solution, at home.

*“All categories of staff can teach tube feeding to mothers. Teaching and training is an allocated task, one nurse a day is allocated to teaching and training. However, all other staff are encouraged to encourage mothers and train as required”. (Nurse, s20)*

In the case of a young child recovering from acute gastrointestinal disease, a mother and a nurse were able to explain to researchers how knowledge sharing in this setting works as a two-way process, enabling the transmission of information about the condition of a young child using the mother as a mediator:

*“I'm feeding the child and changing the nappy, they [nurses] are asking me has my child eaten and how was my child's nappy”. (Mother, s19)*

*“Mothers must show us [nurses] the contents of the nappy before being given another nappy. This is to keep a check on the condition of the child, especially those in the gastro ward”. (Nurse, s20)*

## Discussion

The findings of this descriptive observational study include evidence of formalised policies and nursing practices associated with the presence of mothers in this setting. Explicit rationales for the policy of continuous maternal presence included the need to accommodate mothers, and a belief that mothers’ presence benefited children and assisted with the provision of care. An implicit value underpinning the practices observed was the promotion of “a healthy whole” by keeping the mother and child together. While this is not unique in our experience of paediatric nursing units in a variety of southern and east African countries, we believe it is the first time that nurses have been involved in describing these distinctive nursing practices and articulating the underlying rationales and values.

The rationale for continuous maternal presence identified is distinct from the concepts of family-centred care in European and North American settings which emphasise the importance of nurses involving families in care and partnership and collaboration between families and nurses [3, 11, 12]. Published studies and professional peer conversations from the continent attest to an interest in the topic [2, 8], but highlight the difficulties of applying Western conceptual frameworks in settings which are very different in culture and resources. The practices in this setting may represent a locally developed model of care which intentionally ensure that mothers are never displaced and therefore retain their role as the child’s primary care giver without nurses “taking over”.

Nurses described with clarity how the presence of mothers supported the smooth running of the ward, reducing demands on nurses and contributing to faster healing and recovery for the child. This finding contrasts with other descriptions of maternal involvement in caring for hospitalised children in African settings. In a study of a paediatric ward in Malawi, Phiri and colleagues [2] found evidence that nurses expressed ambivalence regarding involving family members in caring for hospitalised children, feeling that it was wrong to do so in the interests of administrative efficiency, rather than for more idealistic goals associated with partnership or empowerment [2]. Nurses in the Malawian study also expressed concern that the delegation of caring responsibilities to parents placed them in an unclear situation with regard to professional duties and ethical responsibilities [2]. The delegation of tasks such as monitoring patients has also been considered to be problematic or controversial in European and North American settings [39]. Conversely, while valuing the practical assistance of mothers, the nurses in this South African study described a practice of shared care which involved correcting mistakes and overseeing the care provided by mothers. These nurses have taken the decision to delegate responsibility for providing aspects of care, but retain a supportive and supervisory role, within a framework which pursues mothers’ enhanced competence and independence as the ultimate goal.

The expressions of values and philosophy which accompanied nurses’ accounts of their practice described the importance of “a healthy whole” for the child, emphasising that a child in hospital needs a mother to be with them. It is likely that nursing practice in this setting is being shaped by the socio-cultural circumstances and especially the community-oriented caring traditions of both nurses and families in African cultures [3].

### Strengths and limitations

The qualitative methods used in this study effectively supported the identification and description of both explicit and implicit nursing practices and the articulation of richly descriptive accounts of practices, values and rationales. The primary limitations of this study are that it reports on a single site, involving a small sample size and a limited period of observation. However, the study’s aims of describing the nursing practices at one site and for a single point in time were met in full. The extent to which this setting fully corresponds with themes arrived at by the researchers through cross-case study analysis with four other sites in different locations suggests that there may be generalisable elements of an emerging concept that we term ‘Care Through Family’, which remains the focus of further study.

## Conclusions

Practice in this setting represents a promising nurse-led practice innovation that appears to successfully facilitate family involvement in the care of hospitalised children which is contextually specific and shaped by local cultures of caring. This setting is innovative in that it has developed formal policies and protocols associated with this practice and has mobilised resources specifically to facilitate the continuous presence of mothers.

## Data Availability

The datasets used and analysed during the current study, with necessary redactions to protect confidentiality and anonymity, are available from the corresponding author on reasonable request.

## Abbreviation

SRQR: Standards for Reporting Qualitative Research
